# Shank3 deficiency alters midbrain GABAergic neuron morphology, GABAergic markers and synaptic activity in primary striatal neurons

**DOI:** 10.1186/s13041-024-01145-y

**Published:** 2024-09-27

**Authors:** Zuzana Bačová, Bohumila Jurkovičová-Tarabová, Tomáš Havránek, Denisa Mihalj, Veronika Borbélyová, Zdenko Pirnik, Boris Mravec, Daniela Ostatníková, Ján Bakoš

**Affiliations:** 1 Institute of Experimental Endocrinology, Biomedical Research Center, Slovak Academy of Sciences, Dubravska cesta 9, Bratislava, 845 05 Slovakia; 2 Institute of Molecular Physiology and Genetics, Center of Biosciences, Slovak Academy of Sciences, Bratislava, Slovakia; 3 Department of Biology, Faculty of Education, Trnava University, Trnava, Slovakia; 4 Institute of Anatomy, Faculty of Medicine, Comenius University in Bratislava, Bratislava, Slovakia; 5 Institute of Molecular Biomedicine, Faculty of Medicine, Comenius University in Bratislava, Bratislava, Slovakia; 6 Institute of Physiology, Faculty of Medicine, Comenius University in Bratislava, Sasinkova 2, Bratislava, 813 72 Slovakia

**Keywords:** Autism spectrum disorder, Nucleus accumbens, Ventral tegmental area, GABA, *Shank3*, Neurite outgrowth

## Abstract

Abnormalities in gamma-aminobutyric acid (GABA)ergic neurotransmission play a role in the pathogenesis of autism, although the mechanisms responsible for alterations in specific brain regions remain unclear. Deficits in social motivation and interactions are core symptoms of autism, likely due to defects in dopaminergic neural pathways. Therefore, investigating the morphology and functional roles of GABAergic neurons within dopaminergic projection areas could elucidate the underlying etiology of autism. The aim of this study was to (1) compare the morphology and arborization of glutamate decarboxylase (GAD)-positive neurons from the midbrain tegmentum; (2) evaluate synaptic activity in primary neurons from the striatum; and (3) assess GABAergic postsynaptic puncta in the ventral striatum of wild-type (WT) and *Shank3*-deficient mice. We found a significant decrease in the number of short neurites in GAD positive primary neurons from the midbrain tegmentum in *Shank3*-deficient mice. The application of a specific blocker of GABA_A_ receptors (GABA_A_R) revealed significantly increased frequency of spontaneous postsynaptic currents (sPSCs) in *Shank3*-deficient striatal neurons compared to their WT counterparts. The mean absolute amplitude of the events was significantly higher in striatal neurons from *Shank3*-deficient compared to WT mice. We also observed a significant reduction in gephyrin/GABA_A_R *γ2* colocalization in the striatum of adult male *Shank3*-deficient mice. The gene expression of *collybistin* was significantly lower in the *nucleus accumbens* while *gephyrin* and *GABA*_*A*_*R γ2* were lower in the ventral tegmental area (VTA) in male *Shank3-*deficient compared to WT mice. In conclusion, *Shank3* deficiency leads to alterations in GABAergic neurons and impaired GABAergic function in dopaminergic brain areas. These changes may underlie autistic symptoms, and potential interventions modulating GABAergic activity in dopaminergic pathways may represent new treatment modality.

## Introduction

Although alterations in gamma-aminobutyric acid (GABA)ergic inhibitory neurotransmission in autism are intensely studied [1, 2], it remains unclear how and in which brain regions changes in GABAergic neurons contribute to the development of autism. It is relatively well known how GABAergic interneurons proliferate, migrate, and reach their final destinations within the cortical and subcortical regions of the brain [3–5]. Nevertheless, GABAergic neurons are known to be present in the classical dopaminergic brain regions as well. For example, the ventral tegmental area (VTA) is composed of more than just dopamine producing cells, as approximately a third of the number of cells in the VTA are GABAergic neurons [6]. Besides local interneuron functions, GABAergic neurons from the VTA extend their projections to the striatum, particularly the *nucleus accumbens* (nAcc), as well as to other subcortical and cortical brain areas [7, 8]. These projections are part of the so-called long reward-associated neural circuits, which participate in the regulation of social interactions and social processing [2, 9]. Alterations in these neural circuits are likely linked to the pathogenesis of autism.

Investigating the morphology and functional roles of GABAergic neurons within dopaminergic projection areas could contribute to understanding the underlying etiology of autism. For this purpose, it is possible to use the well characterized model of SH3 and multiple ankyrin repeat domains 3 *(Shank3)*-deficient mice, which show autistic symptomatology [10, 11]. In our previous studies, we demonstrated that this autism-like model exhibits changes in neuritogenesis in neurons isolated from specific brain regions [12]. We also observed alterations in the markers of GABAergic neurons within dopaminergic areas of the brain [13]. However, it remains unclear how, or if, the morphology of GABAergic neurons in the dopaminergic brain regions is altered in *Shank3* deficient mice. Additionally, it is unknown whether there are any other changes in GABAergic inhibitory neurotransmission within the striatum in autism-like conditions.

Therefore, the aims of this study were to: (1) compare the morphology and arborization of primary glutamate decarboxylase (GAD)-positive primary neurons isolated from the midbrain tegmentum; (2) evaluate synaptic activity in primary neurons isolated from the striatum; and (3) assess GABAergic postsynaptic puncta in the ventral striatum (corresponding to the nAcc) of wild-type (WT) and *Shank3*-deficient mice. Additionally, gene expression of GABA receptor type A gamma subunit (*GABA*_*A*_*R γ2*), *gephyrin* and *collybistin*, postsynaptic proteins important for clustering and anchoring of GABA_A_R at inhibitory synapses in the VTA and nAcc were evaluated in adult *Shank3-*deficient mice.

## Materials and methods

### Animals

In our experiments we used WT (C57BL/6J) and homozygous knockout (*Shank3*^*−/−*^ here also called *Shank3*-deficient) mice generated by mating of *Shank3B* heterozygous mice (B6.129-*Shank3*^tm2Gfng/J^) with PDZ depletion (exons 13 to 16) [10], obtained from the Jackson Laboratory (Stock No: 017688). Animals were kept on standard water supply and pelleted diet *ad libitum*. All experimental procedures were approved by the Ethical Committee of the Institute of Pathophysiology (07/2017/SKU11016), Comenius University, Bratislava, and have been conducted according to the European Union (EU) Directive 2010/63/EU and Slovak legislation.

### Preparation of primary neurons

Primary neuronal cells from the striatal and tegmental area of WT and *Shank3*^−/−^ mice (2–4 per group) were isolated on the first postnatal day (P0) according to the protocol by Reichova et al. [12]. Cells were planted into 24-well plates with individual 12 mm round poly-D-lysin pre-coated cover slips (10 μg/ml; Sigma-Aldrich, Germany) and incubated under standard condition (37 °C and 5% CO_2_) in neuron-selective-growth medium (Neurobasal A; enriched with 2% B27 supplement (Invitrogen, USA); 100 U/ml penicillin; 100 U/ml streptomycin; 2 mM L-glutamine (all Gibco, USA)) for 5 days. Subsequently, 50% of the medium volume was replaced every two days.

### Morphological analysis

Evaluation of morphological changes was performed on different days in vitro (DIV). On a given day, cells were fixed with 4% paraformaldehyde, pH 7.4 for 20 min at room temperature (RT). Coverslips were gently washed 3 times with ice-cold PBS and blocked for 30 min at RT in normal goat serum (3% NGS in PBS) with presence of 0.1% Triton X-100. Microtubule associated protein 2 (MAP2) and glutamate decarboxylase (GAD) 65/67 were detected with primary antibodies for 120 min at RT (concentration and source are presented in Table 1). After washing with ice-cold PBS (3 times) cells were incubated with corresponding fluorescent secondary antibody diluted in PBS (Table 2) for 60 min at RT. Cell nuclei were stained with 300 nM DAPI (4′,6-diamidino-2-phenylindole; Thermo Fisher Scientific, Germany) for 2 min.

Fluorescent images from two coverslips per animal were captured using an Olympus BX63 microscope at 20x magnification, maintaining consistent detection limits and utilizing automatic deconvolution. FIJI/IMAGE J software was used to evaluate at least 10 areas per coverslip. Cells that were immunopositively stained for both MAP2 and GAD65/67 were identified as GABAergic neurons. Dendritic arborization was analyzed using the Neuroanatomy/Sholl analysis plugin, which measured the number of dendrite intersections with concentric circles at 1 μm intervals from the soma center up to 150 μm.

**Table 1:** Primary antibodies

Name	Host species	Dilution	Method	Product number
anti-GABA_A_R γ2	Chicken	1:500	IHC	224,006, Synaptic System, Germany
anti-GAD65/67	Rabbit	1:500	ICC	ab11070; Abcam, UK
anti-GEPHYRIN	Rabbit	1:500	IHC	pa5-29036, Thermo Fisher Scientific, Germany
anti-MAP2	Mouse	1:2000	ICC	M4403; Sigma-Aldrich, Germany

**Table 2:** Fluorescent secondary antibodies

Name	Host species	Dilution	RRID
**anti-mouse** Alexa Fluor 488	Goat	1:500	A-11,001; Thermo Fisher Scientific, Germany
**anti-rabbit** Alexa Fluor 555	Goat	1:500	A-21,428; Thermo Fisher Scientific, Germany
**anti-chicken** Alexa Fluor 647	Goat	1:500	A-21,449; Thermo Fisher Scientific, Germany

### Spontaneous postsynaptic currents (sPSCs) in striatal neurons

Recordings were performed in a whole-cell configuration of the voltage-clamp at -70 mV using a HEKA EPC10 amplifier (HEKA Electronics). Acquisition and analysis were performed using Patchmaster v90.2. The input resistance and capacity transients were compensated by up to 70% with in-built circuits of the EPC 10 amplifier. Data were acquired at 10 kHz and filtered at 2,4 kHz. sPSCs were recorded in primary culture of striatal neurons on DIV 9–12. The extracellular solution used in the experiments contained (in millimolar): NaCl 127; KCl 2.5, CaCl_2_ 2; MgCl_2_ 1; HEPES 10; glucose 12; pH 7.4 (adjusted with NaOH). Patch pipettes had a resistance ranging from 2.8 MΩ to 3.5 MΩ when filled with a solution containing (in millimolar): CsCl 115; Mg-ATP 3; Na-GTP 0.5; TEACl 10; HEPES 25; EGTA 0.5; pH 7.2 (adjusted with CsOH). The osmolarity of the intracellular solution was approximately 300 mOsmol/L. During the recording, the specific antagonist of GABA_A_ receptors, bicuculline (BIC) in 10 μM concentration was applied using a gravity flow perfusion system. Specific glutamate receptor blockers, 6-cyano-7- nitroquinoxaline-2,3-dione (CNQX, 10 μM) and D-2-amino-5-phosphonopentanoic acid (D-AP5, 20 μM) were applied to assess the character of the remaining sPSCs. Recorded sPSCs were analyzed offline using automatic detecting software Easy Electrophysiology and subsequently manually checked for accuracy. The detection threshold amplitude was set at 10 pA.

### Quantitative real-time PCR

Brain tissues isolated from adult male mice (*n* = 6/genotype) were used to analyze the expression of postsynaptic proteins and GABA_A_R γ2. Animals were sacrificed by decapitation and the brains were quickly removed from the skull; isolated tissues of nAcc and VTA were frozen on dry ice and stored at -70 °C. Total RNA was isolated by a phenol–chloroform method using TRI-reagent (MRC, Germany) according to the manufacturer’s protocol. The reverse transcription procedure was carried out using the High-Capacity cDNA Reverse Transcription Kit (Thermo Fisher Scientific, Germany). qRT-PCR was processed using Power SYBR^®^ Green PCR Master Mix (Thermo Fisher Scientific, Germany) on QuantStudio 5 thermocycler (Thermo Fisher Scientific, Germany). The 2^−ΔΔCt^ value of each sample was calculated using *Gapdh* or *S18* as reference control genes (sequences of primers in Table 3).

**Table 3:** List of primer sequences used in this study. GABA_A_R γ2 – gama 2 subunit of the GABA_A_receptor, S18 – small ribosomal subunit, gapdh – glyceraldehyde 3-phosphate dehydrogenase

Name	Primers	Gene Bank	References
Collybistin	Fw: CAAGGAAACGGAAGAAGTGC	NM_001290385.1	[14]
	Rv: GGGCAGAGTTGACACCTTTC		
GABA_A_R γ2	Fw: ACTTCTGGTGACTATGTGGTGAT	NM_008073.4	[15]
	Rv: GGCAGGAACAGCATCCTTATTG		
Gapdh	Fw: CGGTGCTGAGTATGTCGTGGAGTC	NM_001289726.1	[16]
	Rv: CTTTTGGCTCCACCCTTCAAGTG		
Gephyrin	Fw: GACAGAGCAGTACGTGGAACTTCA	NM_145965.2	[17]
	Rv: GTCACCATCATAGCCGTCCAA		
S18	Fw: CGCCGCTAGAGGTGAAATTC	NR_003278.3	[18]
	Rv: TTGGCAAATGCTTTCGCTC		

### Immunohistochemistry

For the determination of postsynaptic sites in the striatum of adult male WT and *Shank3*^*−/−*^ mice (*n* = 6 animals/group), we investigated the presence of GEPHYRIN and GABA_A_R γ2 colocalization. After the perfusion with 4% paraformaldehyde, pH 7.4, brains were stored overnight in the same fixative and then sectioned on the cryostat (CM1950, Leica Microsystems GmbH, Germany) at a thickness of 30 μm. Sections were temporarily stored (4 °C) in 24 well plates in PBS with 0.01% sodium azide. For immunohistochemistry, free-floating sections were blocked with 3% goat serum (NGS) and 0.01% Triton X-100 in PBS. After 1 h (RT), sections were incubated with a combination of primary antibodies: anti-GEPHYRIN and anti- GABA_A_R γ2 (Table 1) in PBS at 4 °C overnight. After washing with cold PBS (5 min/ 3times), sections were incubated with corresponding secondary antibodies (Table 2) diluted in PBS for 1.5 h at room temperature. Subsequent sections were rinsed with PBS (5 min / 3times), and 300 nM DAPI was added to stain the nuclei. After final PBS washing, sections were mounted on glass slides with Fluoromount-G (Sigma-Aldrich, Germany). GEPHYRIN- and GABA_A_R γ2 -stained sections were imaged by a Nikon confocal microscope (Nikon ECLIPSE Ti-E, A1R+; Netherlands). Free-floating sections (30 μm thick) were imaged at high magnification (40x objective, numerical aperture 1.3; resolution 1024 × 1024 pixels) at 0.5 μm steps. Quantification of colocalization between GEPHYRIN and GABA_A_R γ2 was performed using the Fiji/ImageJ plugin ComDet v0.4.1 [19]. In brief, particles were detected from three different areas of the nAcc in both 555 and 647 channels independently with an approximate size of 3 pixels and an intensity threshold of 3 SD. Colocalization was determined based on the maximum distance of 1 pixel between particles.

### Statistical analysis

Statistical analyses were performed using GraphPad Prism 10.2.3. The data for gene expressions, morphology analysis and colocalizations were first tested for normal distribution using the Shapiro–Wilk test. Comparisons of relative gene expression were performed using the Mann-Whitney non- parametric test. Two-way ANOVA (genotype, arborization) was used for morphological changes analysis, with Sidac test as a *post hoc*. For a colocalization evaluation, data points that fell below Q1–1.5 * IQR or above Q3 + 1.5 * IQR were considered outliers and were therefore excluded from the analysis. Changes in colocalization of GEPHYRIN and GABA_A_R γ2 postsynaptic puncta were evaluated by Student T test. For electrophysiology measurements, outliers were identified using ROUT method. Cleared data were subsequently tested for normality using D`Agostino & Pearson test and a comparison of data for two groups was performed by Mann-Whitney non-parametric test. Results are expressed as the mean ± SEM. The value of *p* < 0.05 was considered statistically significant.

## Results

**Fig. 1:**
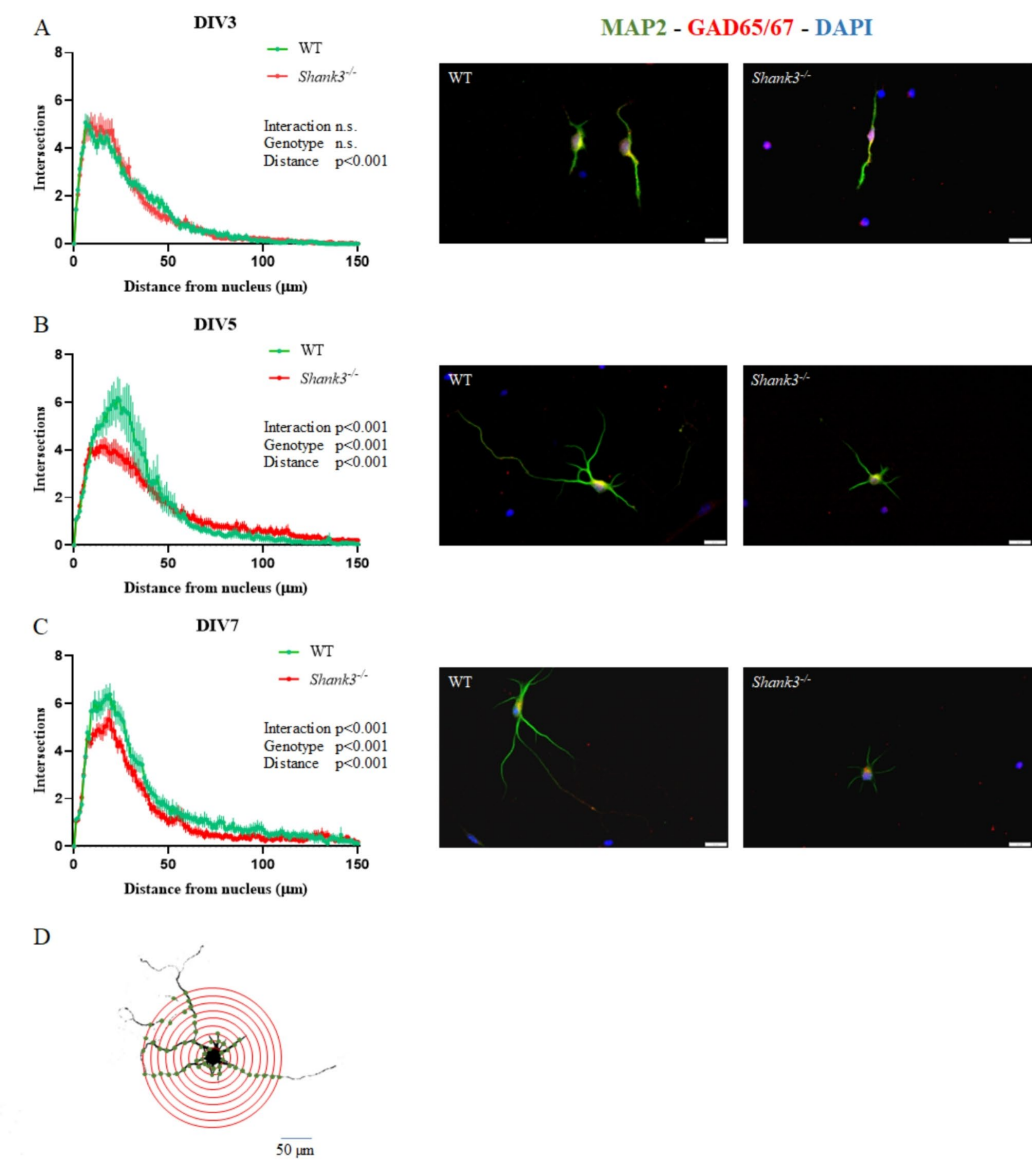
Dendritic arborization in primary glutamate decarboxylase (GAD) positive tegmental neurons isolated from WT and *Shank3*-deficient mice. Neurons were maintained in neuron-selective-growth medium and stained on 3rd, 5th and 7th day in vitro (DIV3, DIV5 and DIV7). Microtubule associated protein 2 (MAP2) served as a marker for neurons, whereas GAD65/67 was utilized to specifically identify GABAergic neurons. Cell nuclei were stained with DAPI. The arborization of the dendritic tree was assessed using Sholl analysis. The number of dendrite intersections (mean ± SEM) for various concentric circles are represented on graphs on DIV3 (**A**, *n* = 50 neurons/genotype), DIV5 (**B**, *n* = 40/ neurons/genotype) and DIV7 (**C**, *n* = 40 neurons/genotype) with representative images of neurons labelled for MAP2 (green), GAD65/67 (red), and DAPI (blue). Representative image (**D**) shows a neuron in binary black and white display with concentric circles. Statistical differences between groups were determined by two-way ANOVA for factors (1) genotype and (2) distance from the cell nucleus. WT - wild type

Primary neuronal cultures isolated from midbrains of WT and *Shank3-*deficient mice were used to evaluate morphological changes in GAD-positive neurons on days 3, 5, and 7 in vitro. Neurite number and branching were assessed using Sholl analysis. Two-way ANOVA was performed for the independent factors (1) genotype and (2) distance from the cell nucleus, separately at each time point. On the DIV3, two-way ANOVA showed no significant effects of factor genotype, but neurite arborization was clearly different (F_(150, 14797)_ = 112.5; p˂0.001) depending on the distance from the nucleus (Fig. 1). On DIV5, statistical analysis revealed significant interaction between tested factors (F_(150, 11778)_ = 2.072; p˂0.001). Significant effects of factors genotype (F_(1, 11778)_ = 12.88; p˂0.001) and distance (F_(150, 11778)_ = 39.87; p˂0.001) were observed. Based on Sidac post hoc test, neurons isolated from *Shank3-*deficient mice exhibited a significantly lower number of shorter neurites within specific distance ranges (19–30 μm). Simultaneously, it was observed that at larger distances from the nucleus, neurite arborization was increased in neurons isolated from *Shank3-*deficient compared to WT mice. A similar significant trend was also observed on DIV7 where there were significant effects of the genotype (F_(1, 11778)_ = 206.6; p˂0.001), distance from the nucleus (F_(150, 11778)_ = 100.7; p˂0.001), as well as the interaction of these factors (F_(150, 11778)_ = 1.35; p˂0.001).

**Fig. 2:**
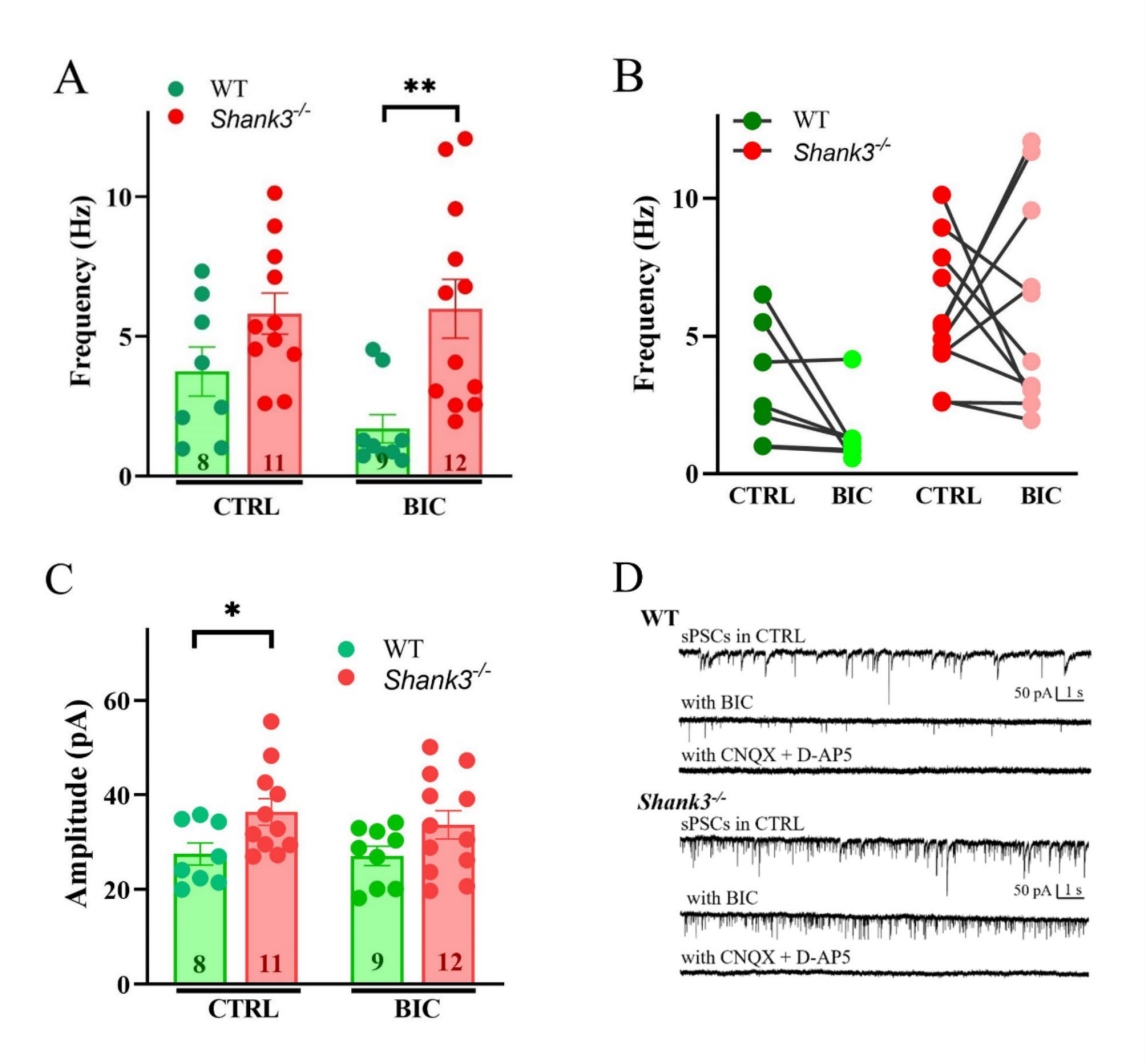
Spontaneous postsynaptic currents (sPSCs) in striatal neurons from wild-type and *Shank3*-deficient mice. (**A**) Averaged frequencies of sPSCs recorded at -70 mV in control extracellular solution (CTRL) and under bicuculline (BIC) treatment in WT and *Shank3*-deficient mice neurons. Data are presented as mean ± SEM (**p < 0.01). (**B**) Change of sPSCs frequency values for individual WT (*n* = 7) and *Shank3*-deficient (*n* = 11) neurons in control conditions and with the application of BIC. (**C**) Averaged absolute amplitude of sPSC events from WT and *Shank3*-deficient neurons in control conditions and with the application of BIC (*p < 0.05). (**D**) Representative sPSC traces recorded from WT and *Shank3*-deficient neurons in control extracellular solution, with the application of BIC and following application of the specific glutamate receptor blockers CNQX (10 μM) and D-AP5 (20 μM)

To determine whether changes in neurite outgrowth from midbrain GABAergic neurons have functional consequences for spontaneous postsynaptic activity in the projection area, we employed the isolated primary neurons from the striatum of WT and *Shank3-*deficient mice. sPSCs recorded at -70 mV in control conditions showed marked although non-significant increased frequency in neurons from *Shank3-*deficient mice compared to WT mice neurons (Fig. 2A). BIC treatment revealed a portion of spontaneous postsynaptic currents indicating GABAergic activity (Fig. 2D). sPSCs with BIC application were notably increased in *Shank3-*deficient striatal neurons compared to WT counterparts (Fig. 2A, B). The mean absolute amplitude of the events was significantly higher in striatal neurons from *Shank3*-deficient mice compared to WT when recorded under control conditions (Fig. 2C). The application of BIC had no significant effect on the mean event amplitude neither in WT neurons nor in *Shank3-*deficient mice neurons.

**Fig. 3:**
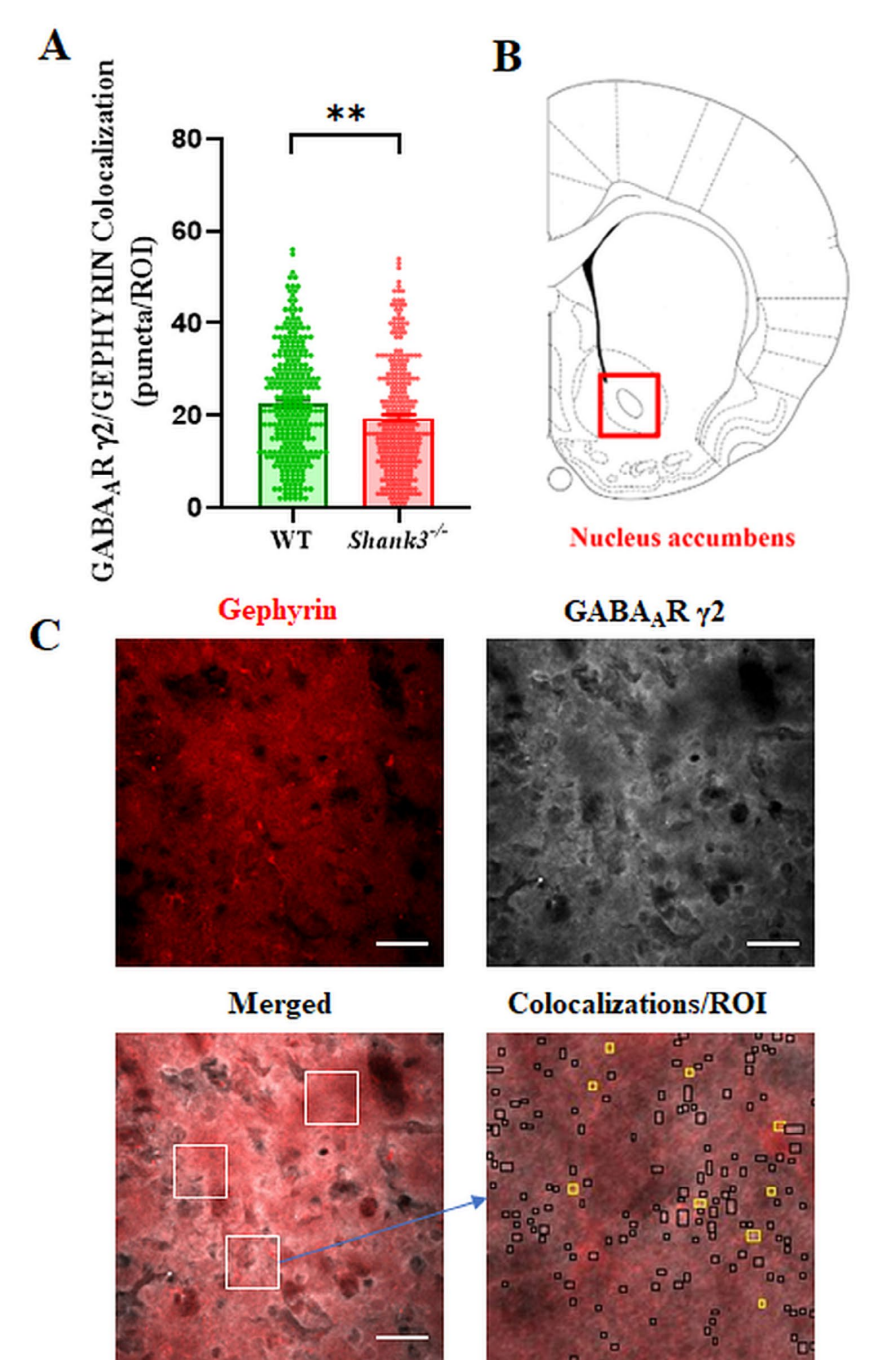
Effect of *Shank3* deficiency on the colocalization of GEPHYRIN/ GABA_A_R γ2 postsynaptic puncta in the *nucleus accumbens*. **A**) Statistical significance between medians of colocalized particles (ROI = 294/genotype) was determined using the Student T test (**p < 0.01). Number of mice: *N* = 6/genotype. Data are presented as mean ± SEM (**B**) Schematic representation of the chosen region (*nucleus accumbens*) and (**C**) confocal microscopy image (40x magnification) showing immunoreactivity to GEPHYRIN (red) and GABA_A_R γ2 (white), as well as image of their colocalization (yellow sqaures) in coronal brain slices. Scale bars represent 50 μm; white box (60 × 60 μm)

**Fig. 4:**
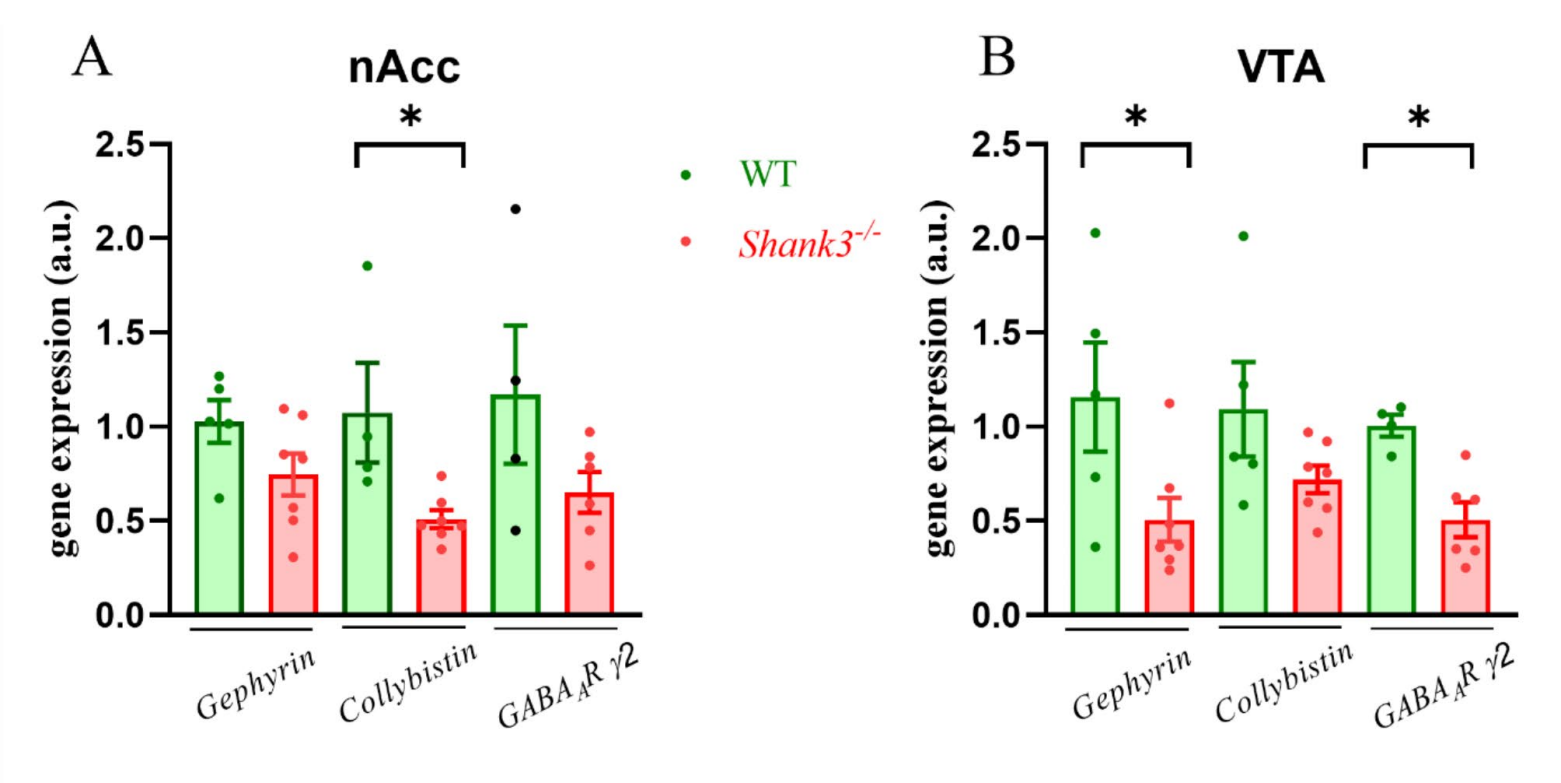
Changes in the gene expression levels of postsynaptic proteins and GABA receptor subunit in the *nucleus accumbens* (nAcc) and ventral tegmental area (VTA) isolated from WT and *Shank3*-deficient male mice. Graphs show relative mRNA expressions. Figures represent relative changes compared to the WT group calculated by 2^−∆∆Ct^ method. *Glyceraldehyde 3-phosphate dehydrogenase* or *S18* were selected as the reference genes. Column scatter dot plot represents values of individual samples and bar represents means ± SEM (*n* = 4–6). Significantly different values are marked with *p < 0.05, Mann-Whitney, WT - wild type. GABA_A_R γ2 – gamma 2 subunit of the GABA_A_ receptor, S18 – small ribosomal subunit

In relation to inhibitory neurotransmission, we conducted an analysis of the colocalization of GABA postsynaptic markers in the nAcc of adult WT and *Shank3-*deficient mice. A significant reduction (Student T test, p˂0.01, T = 3.104, df = 521) in the colocalization of GEPHYRIN/GABA_A_R γ2 postsynaptic puncta in the striatum of *Shank3*-deficient compared to WT mice was revealed (Fig. 3A). To assess whether alterations in GABAergic postsynaptic puncta in the nAcc of *Shank3-*deficient mice are associated with specific GABA_A_R related postsynaptic proteins, we analyzed *gephyrin*, *collybistin* and *GABA*_*A*_*R γ2* gene expression in the VTA and nAcc. The gene expression of *collybistin* was significantly (Mann-Whitney, p˂0.05, U = 1, df = 9) decreased in the nAcc and *gephyrin* in the VTA (Mann-Whitney, p˂0.05, U = 5, df = 10) in *Shank3* deficient compared to WT mice (Fig. 4). Gene expression of *GABA*_*A*_*R γ2* was significantly decreased (Mann-Whitney, p˂0.01, U = 1, df = 8) in the VTA of *Shank3-*deficient compared to WT mice.

## Discussion

In the present study, we observed significant changes in the neurite outgrowth and branching of GAD-positive primary neurons isolated from the tegmental area of the midbrain in *Shank3*-deficient mice. We also detected a notable reduction of GABAergic postsynaptic puncta and selected postsynaptic proteins in the dopaminergic brain regions of *Shank3*-deficient mice, which was accompanied by enhanced spontaneous postsynaptic activity in isolated striatal neurons.

The finding that *Shank3*-deficient mice exhibit altered arborization of GABAergic neurons isolated from the midbrain suggests changes in neurite growth and synaptic connections, potentially affecting signal processing in dopaminergic brain regions under autism-like conditions. It is well known that abnormal connectivity in dopaminergic pathways contributes to the pathogenesis of autism [3, 20]. Moreover, recent studies point to long-range connections of GABAergic neurons that intertwine with VTA dopaminergic neurons [21, 22]. It is therefore possible that insufficient growth of midbrain GABAergic neurons results in functional striatal abnormalities in *Shank3*-deficient mice. In our study, we surprisingly found a significantly higher amplitude of sPSCs in striatal neurons from *Shank3-*deficient mice when recorded under control conditions. The application of BIC had no significant effect on the amplitude neither in WT neurons nor in *Shank3*-deficient mice neurons. Since SHANK3 is localized in postsynaptic endings, it is possible that these signals are the result of tonic disinhibition of striatal neurons in vitro as these neurons are regulated by dopamine release under in vivo conditions [23]. It is important to note that GABAergic neurons from the VTA are believed to project to nAcc interneurons [24]. A limitation of our approach is that we did not distinguish the phenotype of the evaluated neurons, despite the known presence of medium spiny neurons. Moreover, the consequences of *Shank3*-deficiency-related synaptopathy in striatal neurons can be cell-specific [25]. Most studies indicate reduced inhibitory currents or altered excitatory/inhibitory balance in the striatum in autistic model tissues [10, 26, 27]. However, it is important to note, that GABAergic conductance in the striatum exhibits considerable heterogeneity and variation and may be even more pronounced under pathological conditions [28].

In this study, the observed increase in the frequency of sPSCs under BIC in striatal neurons isolated from *Shank3*-deficient mice may suggest that there are more excitatory postsynaptic currents occurring spontaneously. Although, in our study, these are isolated primary striatal neurons and not the entire tissue, which means we cannot determine the precise presynaptic cause of these observations, it is possible that the reduction of inhibitory input allows for more frequent firing of excitatory neurons. sPSCs in striatal cells from *Shank3*-deficient mice may be related to the observed decrease in GEPHYRIN/GABA_A_R γ2 colocalization in the nAcc. Gephyrin is a key scaffolding protein essential for stabilizing GABA receptors at inhibitory synapses [29]. Reduced colocalization of GEPHYRIN/GABA_A_R could lead to a compensatory increase in excitatory synaptic activity [30]. This suggests that the structural changes at the synapse due to reduced GEPHYRIN and GABA_A_R interaction may drive functional alterations in synaptic transmission. Another indicator of GABAergic synapse remodeling in the striatum is the decreased expression of *collybistin* in the nAcc observed in this study. Collybistin interacts with gephyrin and is involved in the clustering of GABA_A_Rs [31]. In autistic conditions, the disassembly of these complexes has been suggested [32, 33]. Notably, bilateral GABAergic projections from the VTA to the striatum, as well as from the striatum to the VTA, are implicated in pathogenesis of autism. Thus, the remodeling of GABAergic synapses in these areas may be induced by *Shank3* deficiency. Other studies have shown an abnormal striatal neuronal morphology in *Shank3*-deficient mice [10]. These neurons are involved in microcircuits within the striatum and also participate in long-range cortical projections to and from the midbrain [34]. Although Peca et al. [10] noted an increase in the complexity of dendritic arborization in *Shank3*-deficient mice using Sholl analysis, our findings indicate, on the contrary, a decrease in the number of short neurites, especially in GAD positive neurons in the midbrain tegmentum. Other studies have also found that SHANK3 knockdown in human induced pluripotent stem cells can lead to a decrease in dendritic arborization [35]. Moreover, shortened neurite outgrowth in primary hippocampal neurons isolated from *Shank3*-deficient mice was previously demonstrated [12].

In conclusion, *Shank3* deficiency seems to cause alterations in GABAergic neurons, leading to impaired GABAergic function, as observed in two other *Shank3*-deficient models [36]. Our electrophysiological data also confirm that the inhibition of GABA_A_Rs leads to increased synaptic activity of striatal neurons from *Shank3*-deficient mice, highlighting the well-known excitation/inhibition imbalance in the context of autism. This interpretation is further supported by the reduction of GABAergic postsynaptic puncta in the striatum of *Shank3*-deficient mice in this study, which corresponds to previous findings observed in the piriform cortex [14]. Similarly, human postmortem ASD samples revealed reduced numbers of cortical GABAergic interneurons and altered branching, correlating with core autism symptoms such as repetitive behaviors and motor stereotypies [37]. Morphological and functional properties of GABAergic neurons that originate from the midbrain and project to the striatum require further investigation, particularly in the context of behavioral changes associated with autism.

## Data Availability

The data that support the findings of this study are available from the corresponding author upon reasonable request.
